# The reasons for the epilepsy treatment gap in Kilifi, Kenya: Using formative research to identify interventions to improve adherence to antiepileptic drugs

**DOI:** 10.1016/j.yebeh.2012.07.009

**Published:** 2012-12

**Authors:** Julie A. Carter, Catherine S. Molyneux, Caroline K. Mbuba, Jo Jenkins, Charles R.J.C. Newton, Sally D. Hartley

**Affiliations:** a Centre for International Health and Development, Institute of Child Health, London, UK; b KEMRI/Wellcome Trust Research Programme, Centre for Geographic Medicine Research (Coast), Kilifi, Kenya; c Neurosciences Unit, Institute of Child Health, University College London, London, UK; d Clinical Research Unit, London School of Hygiene and Tropical Medicine, London, UK; e Department of Psychiatry, University of Oxford, Oxford, UK; f Faculty of Health, University of East Anglia, Norwich, UK

**Keywords:** PWE, people with epilepsy, ETG, epilepsy treatment gap, FGDs, focus group discussions, RPCs, resource‐poor countries, AEDs, antiepileptic drugs, CHWs, community health workers, CWE, children with epilepsy, THs, traditional healers, Qualitative research, Epilepsy treatment gap, Adherence, Antiepileptic drugs, Interventions, Kenya

## Abstract

Many people with epilepsy (PWE) in resource‐poor countries do not receive appropriate treatment, a phenomenon referred to as the epilepsy treatment gap (ETG). We conducted a qualitative study to explore the reasons for this gap and to identify possible interventions in Kilifi, Kenya. Focus group discussions (FGDs) were carried out of PWE and their caregivers. Individual interviews were conducted of PWE, their caregivers, traditional healers, community health workers and leaders, nurses and doctors. In addition, a series of workshops was conducted, and four factors contributing to the ETG were identified: 1) lack of knowledge about the causes, treatment and prognosis of epilepsy; 2) inaccessibility to antiepileptic drugs; 3) misconceptions about epilepsy derived from superstitions about its origin; 4) and dissatisfaction with the communication skills of health providers. These data indicated possible interventions: 1) education and support for PWE and their caregivers; 2) communication skills training for health providers; 3) and improved drug provision.

## Highlights

► Many with epilepsy do not receive appropriate treatment; epilepsy treatment gap (ETG). ► Qualitative study exploring reasons for ETG and identifying viable interventions in Kenya. ► ETG can be addressed through development of multifaceted interventions.

## 1 Introduction

Over 69 million people worldwide have epilepsy, of which 62 million live in resource‐poor countries (RPCs) [1]. More than 500 million people are indirectly affected by epilepsy as parents, relatives and friends [2]. The World Bank has prioritized epilepsy as a highly cost-effective condition to treat [3] since relatively inexpensive antiepileptic drugs (AEDs) are very effective in controlling seizures: with up to 75% of those treated will become seizure free [4]. Despite this fact, over 90% of the people with epilepsy (PWE) in RPCs do not receive appropriate treatment for their condition [5–7], a phenomenon that has been called the ‘Epilepsy Treatment Gap (ETG)’. The International League Against Epilepsy (ILAE) has defined ETG as the difference between the number of people with active epilepsy and the number of people whose seizures are being appropriately treated [8].

In response to the diverse factors involved in the effective treatment of PWE in RPCs, several researchers have tried to instigate affordable community-based interventions. Previous interventions at community the level have met with some success in Nakuru, Kenya [9], Ecuador [10], Malawi [11] and India [12,13]. Recent directives from the World Health Organization (WHO) state that health care delivery will increasingly have to move away from acute illness episodes and concentrate more on treatment adherence, client self-management and quality-of-life issues [14].

The characteristic of most successful interventions is integration within community health care delivery and community involvement in the planning and implementation processes. Community-based interventions have been used with some success in several conditions. For example, in Nigeria, community-designed and community‐administered treatment programs have been successful in the control of onchocerciasis [15], and in Bangladesh, community health workers (CHWs) have successfully identified tuberculosis (TB) and increased adherence to treatment in their own villages [16].

Most commentators highlight the need for good anthropological or community-based data on practices and perceptions for behavioral interventions to succeed [17]. In Kilifi, there is some evidence that bio-medical treatment regimens for children with epilepsy (CWE) are in conflict with local perceptions [18]. Parents may have a ‘health’ versus ‘sickness’ model that influences their perceptions of treatment. Occasional seizures may be placed in the ‘health’ sphere, making the recommendation of regular and continued medication illogical for what is perceived to be a generally healthy child. If regular seizures persist beyond a certain age, the child may be placed in the ‘sickness’ sphere, suggesting that the child is incurable and treatment attempts are futile. El Sharkawy and colleagues also found that belief in ‘external’ causes (such as witchcraft, contact with certain animals/birds) of epilepsy was commonly held, and that treatment choices favored external treatments, such as wearing charms or pouring liquid on the child's body [18]. These findings are similar to those of Hausmann-Muela and colleagues, who investigated what they termed as ‘medical syncretism’ in Tanzania [19]. They found that in the case of malaria, biomedical knowledge is blended with indigenous concepts [19]. These examples fall under Helman's description of ‘externalising’ belief systems, which concentrate on etiologies arising outside of the sick child's body [20]. If the cause of an illness is believed to be found in the natural, social or supernatural worlds, biomedicine may be sought for symptomatic relief only, but the cure and explanations about the causality are sought from traditional healers (THs) [20].

Identifying the cultural context, values, beliefs and community norms of target groups through qualitative research is the key to the design and implementation of successful interventions [21]. Akogun and colleagues comment that their experience of working with a Nigerian community to implement a behavioral intervention suggests that within certain parameters, the structure of interventions may be less important than the process through which they are introduced, in terms of acceptability and sustainability [15]. Building upon the work of El Sharkawy and colleagues [18], we aimed to carry out a qualitative investigation to develop alternative intervention strategies based on the expressed needs and perceptions of the stakeholders. While El Sharkawy and colleagues focused on the effect of attitudes and practices on service utilization for children with epilepsy [18], we specifically conducted this descriptive research as part of preliminary work to develop an intervention to address the ETG in both adults and children with epilepsy, within the broader community of Kilifi District, including traditional medicine. Our research questions were:

1. What are the stakeholders' (PWE, their families and service providers) attitudes and beliefs relating to PWE and how do they affect the utilization of the traditional and biomedical services offered to this population group?
2. What are the specific needs of PWE and their families?
3. Using techniques such as participatory workshops, can a consensus be reached with key community members on an intervention that will reduce the ETG and improve the quality of life of PWE in Kilifi?

## 2 Methods

### 2.1 Study setting

This qualitative study was conducted in the Kilifi Health Demographic Surveillance System (KHDSS) located in Kilifi District on the coast of Kenya. It comprises 15 locations with 40 sub-locations sub-divided into 187 enumeration zones that can be easily located using digital maps of homesteads. It covers an estimated area of 891 km^2^ with 233,880 residents in 28,000 homesteads. The residents are mainly Mijikenda, a Bantu grouping of nine tribes with Giriama (45%), Chonyi (33%) and Kauma (11%) dominating. The average per capita income is about Ksh.700 (10 US dollars) per month, and about 55% of the population is considered poor. The majority (80%) depend on subsistence farming which is limited by the low productivity of the land since only 19% of the land is arable. Literacy levels are low: only 45% of the people can read and write [22].

### 2.2 Sample

Families from different educational backgrounds and with different experiences of AED treatment were purposively selected using the baseline information recorded on the KHDSS. The criteria for selection are outlined in Table 1. The target age group was anyone with epilepsy — adult or child. The selection criteria were treatment status, educational background and severity of epilepsy. Once appropriate participants were identified, convenience sampling was used, influenced by willingness to participate, geographical distribution and time constraints.

Service providers (CHWs, in the case of the FGDs) were identified from lists of active community health worker groups held by the local Ministry of Health. Community health workers are members from the community who have volunteered or have been selected by the village leaders to receive extra training about health matters. They often do this on a voluntary basis but receive some payment, for example, when attending seminars on health. Initial pools of participants for individual interviews and participatory workshops were elicited from FGD participants, using a snowballing technique. Final selection and participation were again based on convenience and voluntary informed consent. The composition of key informant groups is described in Table 2.

### 2.3 Procedure

**Fig. 1:**
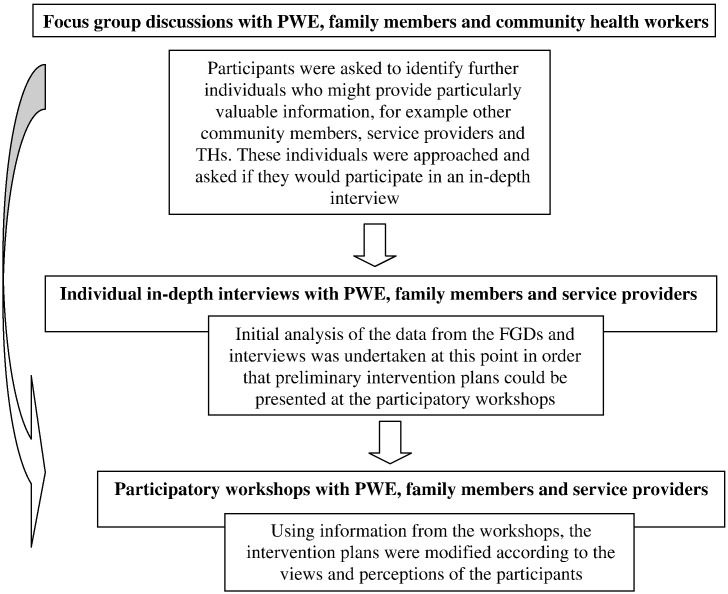
Progress of the study, and how the findings informed intervention development.

The study included a combination of strategies for data collection – FGDs, individual interviews and participatory workshops – to enhance the validity of the results and provide material for triangulation. The study was conducted from 2004 to 2006. The progress of the study is illustrated in Fig. 1.

First, FGDs were held with adults with epilepsy, CWE, parents of CWE, other family members of CWE and CHWs. Efforts were made to ensure that groups of CWE and of parents covered the spectrum of severity of disease, educational levels and exposure to AED treatment. Such purposive convenience sampling is appropriate for research designs such as this, which aim to explore the range – rather than a representative cross section – of views and perspectives. Excluded from the discussions were adults or CWE who considered themselves or were considered by family members to be too ill to participate.

Focus group discussions elicited data relating to the four research questions:

• Experiences and beliefs about epilepsy in the household and in the community (particularly in schools, marriage and workplace);
• How the above relates to the quality of life, parental satisfaction and depression;
• The range of treatments available for children, and the pros and cons of different types and sources of treatment, including experiences of and views about AEDs;
• Perspectives of the principal needs of PWE, of the barriers to meeting these needs, and – where appropriate – of locally applicable interventions to improve access to AEDs.

Individual in-depth interviews were organized to explore issues arising in more detail, including PWE, their families and service providers. The aim was to identify providers working in a wide range of settings who have substantial experience of managing epilepsy. It was hoped that discussions with THs would bring opportunities to present locally-held ideas about causation and preventive treatment [4], so particular attention was given to this group and the possible necessity for increasing the sample size to access further data. We found it difficult to bring THs together in a FGD setting due to their superstitions about KEMRI and about each other. Thus, we interviewed them individually. Epilepsy may offer a particularly good basis for cooperation between biomedical and traditional medicine, particularly when neither may satisfy the needs of the patients alone [8].

Individual interviews with these participants focused on:

• Experiences of and views about the range of treatments available for children;
• The extent and nature of the ETG in Kilifi, and the range of factors contributing to these gaps (including regulatory and supply factors, and information, education and communication issues at provider, patient and caretaker levels);
• Relevant and sustainable interventions to reduce the ETG, and – if appropriate – the inputs that would be required to improve access to AEDs.

### 2.4 Participatory workshops

Drawing on the information gathered above, a series of workshops was arranged. The workshops aimed to negotiate a realistic and sustainable intervention with key stakeholders to improve the ETG in the Kilifi District. Key stakeholders included PWE, their families and service providers from the District. It was felt that these groups were key in family decision making about epilepsy and its treatment, but it is important to note that community leaders (e.g. religious leaders) could have been included and, thus, is a limitation of the current study.

We anticipated that users and providers would have differences of opinion at the outset, and that the intervention would require processes of negotiation. To ensure that all viewpoints were heard and incorporated, separate one-day workshops were held with representatives from the following groups: CWE and their siblings; parents and grandparents; and service providers (four workshops over four days). During the workshops, initial intervention plans (developed from the FGD and interview data) were presented to the participants to elicit group consensus or normative reaction. The participants were also asked to give specific input about the practicalities of the intervention: length, administered by whom, location, incentives and outcome measures.

Based on the suggestions from the workshops, an education intervention was developed for all stakeholders. It was divided into four parts: the first one for CHWs, second for THs, third for community members and fourth for biomedical providers. The intervention components for the first three groups were similar, but for biomedical providers, it was different (Table 3).

## 3 Ethical considerations

The study was explained to respondents by trained field staff using the local language. Written informed consent was obtained from all study participants. Approval for the study was obtained from the Kenya Medical Research Institute (KEMRI)/National Scientific Ethical Review Committees.

## 4 Analysis

Focus group discussions and interviews were conducted in the languages of the participants, namely, Kigiriama, Kiswahili and English. They were recorded, translated and transcribed. The data were entered onto a NVivo qualitative analysis software (version 9, QSR; Melbourne, Victoria, Australia; http://www.qsrinternational.com/) to enable easy storage, organization and retrieval. Data were analyzed using framework analysis, as described by Ritchie and Spencer [23]. Themes were independently generated from the data by two researchers, and once thematic consensus was reached, all the data were coded. This process served to maximize the rigor and validity of the analysis.

Emerging patterns and relationships were used to generate an initial intervention plan that was grounded in the qualitative data. Two stages of modification then took place. The plan was presented at the participatory workshops, at which modifications were made until a consensus was reached among the participants. Second, the research team made final modifications to the educational element of the intervention following the pilot study.

## 5 Results and discussion

### 5.1 Themes

In using these results to inform the intervention, the findings were focused on two categories: (1) reasons for the ETG and how the intervention could address these issues; (2) intervention content and format. In looking at the reasons for the ETG, five main themes emerged from the data: differing explanations for the causes, treatment and prognosis of epilepsy; the issue of cure; diminished access to and utilization of biomedical health services; poor doctor–patient relationships and communication and access issues for PWE and their families.

#### 5.1.1 Differing explanations for the causes, treatment and prognosis of epilepsy: impact on treatment choice

Among PWE and their families, knowledge about the biomedical causes, treatment and prognosis of epilepsy was minimal. There were multiple causal explanations for the condition. Discussions on the root causes of symptoms suggested that they can be attributed to the natural order (brain insults following diseases such as malaria, accident, perinatal injury, inheritance, caught from contagious urine, drugs):

“*[Epilepsy] is inherited. Some people fear even marrying from a family where people have fits …. They think if you marry from such a family, it is easy to have a child who has it.*” (FGD CHWs)

“*[It was caused by] falling down from the back of the mother. She fell when she was still very young … the cloth loosened and she fell and the back of the head depressed. It all started from then. She fitted up to now.*” (Interview grandmother)

“*When not fitting the person is very fine and like if someone is not asthmatic and you give anti-asthma drugs, they get it so it's the same to the epileptic person, if you give epileptic drugs he will get epilepsy.*” (Interview diviner)

Others ascribed epilepsy to interpersonal issues (bewitchment/genies due to jealousy or arguments; home/marital issues):

“*It is said that it is witchcraft. She was bewitched, that is according to our customs. That is when you will go to a mganga [traditional healer] because you want to untrap them.*” (FGD grandmothers)

“*They say it's [caused by] jealousy from the surrounding people because you have children.*” (Interview father)

However, there were shifting causal explanations, often depending on the perceived type of epilepsy: one grandmother commented “*if it's normal or from God, one can be healed, but if it's due to somebody's wish [i.e. a curse], you will treat in vain*” (FGD grandmothers).

Beliefs inevitably impacted on the type of treatment sought. One man with epilepsy commented that the cause of his condition was “*things that go wrong in the home*”. He explained: “*[A family member] can go to the extent of having sex with a cousin. When they are discovered, it's when a sheep can be slaughtered if not, that's what can bring illnesses.*” (Interview adult with epilepsy). El-Sharkawy and colleagues discussed the differentiation of perceived causes of epilepsy into internal and external phenomena in this community [18]. They found that belief in ‘external causes’ (i.e. requiring an agent independent of the child, such as trauma or witchcraft) was reflected in treatment choices, usually that of traditional medicine [18]. Our findings were similar and reinforce the importance of THs as being one approach within households to the treatment of epilepsy, emphasizing the need to recognize this factor in the design of the intervention:

“*With me, I think they didn't see the illness, that's why they discharged her. Maybe she was bewitched and the machines [at the hospital] can't detect witchcraft.*” (FGD CHWs)

“*We also tell people if they have gone to the hospital and found the condition is not the type to be treated there, they should come back to us.*” (Interview Traditional Healer)

Apart from traditional beliefs, the local churches and mosques also played a role in some people's treatment-seeking behaviors (usually, taking the person to be prayed for). More commonly though, people said they had no idea what caused the condition. Several comments suggested that the transient nature of seizures and less recognizable types of epilepsy added to the confusion about the causality:

“*I don't know [what it is] because it's something that comes just for some time then it goes.*” (Interview CWE)

“*According to her symptoms, I have not believed it is epilepsy because there are signs that come first and then she fits … initially you will see that she has changed and becomes pale, and then she reports about leg pains; then at night she jerks and says ‘my legs are pulled away’ …. So I think in my mind, does an epileptic person have such symptoms? No!*” (Interview mother)

#### 5.1.2 The issue of cure

Adherence to drugs is known to be problematic when regimens are drawn out or where there is rapid decline of symptoms before the completion of the course of drugs. The concept of prolonged drug regimens to control rather than to cure a condition is difficult to understand although with the increasingly widespread use of ARVs, this might be changing:

“*If they take the drugs, they stay for a long time without fitting but they will eventually fit but what we want is that they get cured completely.*” (FGD grandmothers)

“*When they take drugs, instead of getting better then it comes back again, what we want is that they take the drugs – no matter for how long – but when they finish them, the illness is gone.*” (Interview father)

“*There should be drugs which people are sure if they take, they get cured completely.*” (FGD CHWs)

Several people expressed confusion and frustration about where they should go for treatment. Many made comments such as “searching in vain” and “going here and there”, for example:

“*You will find that sometimes you are moving here and there looking for the treatment which can heal your child but in vain. It is not in every place that you go that you can get healed.*” (FGD mothers)

#### 5.1.3 Diminished access to and utilization of biomedical health services

Distance to health facilities as a barrier to biomedical health services was a recurrent theme from both service providers and users:

“*I want to say that taking a child to [the hospital], it takes a long time. If you could bring the drugs here, people would save time and bus fare.*” (FGD CHWs)

“*You have to make sure that you get bus fare before the drugs are finished but if you pass two weeks without drugs because you don't have the fare, then he fits so seriously.*” (FGD grandmothers)

“*The hospital is far away and we don't have transport. We have to find a vehicle to get there.*” (Interview dispensary nurse)

Even for those who had reached the hospital, service was not always assured, and participants complained that they could spend a whole day without seeing a health professional:

“*The bad thing about the hospital service is the delay …. You are sent here and there until whenever you reach where you are supposed to, you have really taken a long time and you lose hope because of the many trips you make.*” (FGD CHWs)

“*Sometimes one spends the whole day [at the hospital] without being served.*” (FGD CHWs)

The other major constraint to utilizing services, as hinted above, was financial restrictions. The recurrent cost of treatment, the effects of diverting limited financial resources from the family's needs to the funding of treatment and the fact that in the Kenyan medical system, an inability to pay means doing without the necessary drugs were all prominent themes, for example:

“*The child could start fitting again or even worse until you run back to the mganga [traditional healer]. He treats him again and the money owed becomes bigger and you have to make sure you have paid fully if you want your child to get better.*” (FGD mothers)

“*This child is sick, so the money that I would use on another project which could support the family I use it for him. I deteriorate because of the one with epilepsy.*” (FGD mothers)

“*The hospital is good but sometimes you go there, you are examined and drugs are prescribed but you need money for those drugs. So if you don't have the money, then you just remain with the illness.*” (FGD CHWs)

These findings highlighted the fact that a successful intervention will have to include factors other than education and information.

#### 5.1.4 Poor doctor–patient relationships and communication

With one or two exceptions, participants generally felt that the doctors they consulted showed little interest or sensitivity and lacked communication skills:

“*Sometimes, just as you have started explaining the problem, the doctor has already finished prescribing the drugs. So you take drugs that you are not confident with at all.*” (FGD CHWs)

“*When the drugs had finished, I didn't understand the doctor, whether I was to go back when the drugs were over. When I went the other day, he asked me why I hadn't gone for more drugs so I told him that I didn't understand him on the first day.*” (Interview mother)

“*I brought my child and was taking him down from my back as the doctor was asking what was wrong. He said he didn't need to see the child and just prescribed drugs. I took the prescription and tore it in two pieces then went to see another doctor and my child was admitted to hospital.*” (Participatory workshop, parents/grandparents)

Lack of understanding of what the doctor was prescribing or the course of treatment he/she was suggesting was a prominent theme. This also extended to knowledge of hospital treatment:

“*When I go with him to the hospital, they take him inside so I don't know what they do to him.*” (FGD mothers)

The lack of understanding was often linked to lack of time and interaction with the doctor, and a feeling that consultations were rushed. A comment from a doctor during an interview suggested that they also feel these pressures: “*Usually there are so many people in the clinic, we don't have time for them all. We are under-resourced.*” (Interview Dr L.)

#### 5.1.5 Other issues for PWE and their families

Many participants described the challenges in the lives of PWE and their families, emphasizing the fact that epilepsy brings a range of other issues: physical, financial, social and cultural. Several families described how epilepsy had affected their children's development:

“*It's [child's name] who is still having that problem, though it's four years since she fitted but her brain still is not normal*” (FGD mothers)

“*She has changed … the brain … the words show you that there is something wrong with her because you will see the words have changed.*” (Interview mother)

“*With my granddaughter you have to feed her. Even toileting has to be assisted.*” (FGD grandmothers)

Perceived and actual risks also meant that some CWE missed out on schooling:

“*My high hopes are that she gets healed so that she can go to school. For I tried taking her to school but the teachers were concerned about how it would be if she falls.*” (Interview father)

“*Some children even want to go to school but they can't because of how they are. They just stay at home.*” (FGD CHWs)

“*Some parents think that because the child has epilepsy, he is not going to learn, maybe he will get the seizures when at school.*” (Interview Dr I.)

Physical injuries sustained during seizures were also reported:

“*When you are cooking you may fall into the fire and lose consciousness. You may get burns.*” (FGD CWE)

“*My granddaughter is missing two of her teeth because she fell in a bad place*.” (FGD grandmothers)

Expressions of hopelessness and despair were common and often accompanied feelings that all potential options had been exhausted, or there were difficulties in obtaining treatment:

“*I am feeling so bad, I don't know how [the epilepsy] will stop.*” (Interview CWE)

“*I went everywhere until I had despaired. I would be given things or advised to do things but on administering, it would worsen. He could stay awake the whole night or take even a whole week fitting.*” (FGD mothers)

“*I try my best to take the child to the hospital for although I have a husband, he is not around, he doesn't come home. I am alone.*” (FGD mothers)

“*You take him to a mganga not at will but out of desperation.*” (FGD mothers)

Several family members of PWE described the desperate measures people may feel driven to:

“*Some people are tired of taking care of that child for the illness has stayed for a long time, so they just leave the child with burns or even still on the fire.*” (FGD grandmothers)

“*You can be very scared, especially when you don't know anything about it. You can run away because whatever you are trying doesn't work.*” (Interview father)

These findings emphasized the need for support to be part of any intervention to address the ETG and the wider issues of quality of life of PWE and their families.

### 5.2 Recommended strategies, content and format for intervention

These data indicated several possible avenues for interventions: first, a lack of understanding about epilepsy and its treatment suggested the necessity of information and education; second, dissatisfaction with the interpersonal skills of service providers suggested communication skills training for those providing epilepsy care; third, problems with accessibility of drugs suggested the need to work with the Ministry of Health to improve drug provision in the community; fourth, the prominent role of traditional healers highlights the need to increase cooperation and dialog with these service providers; and finally, the levels of stress expressed by many participants highlighted the importance of support for PWE and their families.

#### 5.2.1 Education and information

Education and more information emerged as prominent themes, both among the families with epilepsy and the service providers. In particular, information about the symptoms and causes of epilepsy, advice about safety, and, for the service providers, further advice about the treatment.

In the participatory workshops, service providers again emphasized that CHWs, doctors, nurses and public health technicians should also be included in any educational interventions although THs and CHWs suggested that relatives of PWE should be given first priority for education.

Various suggestions for who should provide information and education were given during the participatory workshops. Community health workers were keen to be involved, one commenting “*we are the ones who stay in the community so it would be better if we were involved more*” (Participatory workshop: CHWs/THs). Other participants suggested staff from KEMRI and parents and family members of PWE, supported by advisors from KEMRI, the rationale being that family members are the ones with direct experience of the issue (Participatory workshop: parents/grandparents).

The participatory workshops also dealt with issues such as where an educational intervention should be held and how long it should last. On the former issue, participants in three workshops (parents/grandparents; THs/CHWs; service providers/CHWs) felt that a central place that could easily be reached, such as a church or school, would be the most appropriate location. This would reduce the cost issue (i.e. transport costs) and allow more people to attend because the disorder is everywhere. Other suggestions included a location near health facilities so that participants could make use of them at the same time (Participatory workshop: THs/CHWs) or in people's homes (Participatory workshop: PWE).

Regarding the time any educational intervention should take, a longer-term educational program with regular home visits was favored as it was felt to be new information, which needed time to be digested (Participatory workshop: PWE). In addition, participants commented that breadwinners cannot afford a chunk of time away from the household (Participatory workshop: parents/grandparents; THs/CHWs). Practicalities such as the provision of meals or compensation for time (a packet of corn flour was mentioned) were also raised (Participatory workshop: service providers/CHWs; THs/CHWs; parents/grandparents).

#### 5.2.2 Communication skills training

Participants in all of the participatory workshops felt that people were not given a clear explanation about AEDs, and they generally did not know what was happening with the treatment. There was general agreement that the service provider's interpersonal skills made a difference to the patient's well‐being and to other patients' use of the service, as exemplified by these comments:

“*Sometimes it depends on the person providing the service. Some are human and treat people so well, just the reception can give you hope. But there are those who are so inhuman, they mistreat people.*” (FGD CHWs)

“*It's the reception that people don't know but if you treat them well, you will see other people coming. If you treat somebody well, that is a lamp already to bring more people.*” (Interview APDK)

#### 5.2.3 Increasing accessibility to AEDs

Accessibility was related to both finances and distance to the health facilities:

“*I want to say that taking a child to Kilifi, it takes a long time. So if they could bring the drugs here, we would have saved time and fare.*” (FGD CHWs)

“*The hospital is far and we don't have transport. We have to board vehicles so as to reach there.*” (Interview dispensary nurse)

“*This hospital is good but sometimes you go there, get examined and prescribed for drugs and you need money for those drugs. So if you don't have money, then you just remain with the illness.*” (FGD CHWs)

Several of the service providers stressed that any new venture should be incorporated into the existing health system to ensure sustainability:

“*If it's within the medical wards or the medical out-patients, even if the study runs out, it will have been established within an already running system so it will remain there, it will not die. But if you've got an external thing, it will stop with the study*.” (Interview Dr I.)

#### 5.2.4 Traditional healers (THs)

Traditional healers were often consulted by the PWE and their families and provided explanations for the development of epilepsy that the PWE and their families could understand and found more acceptable than the biomedical explanations. Most people found the interaction with traditional healers to be very positive:

“*When I am at this place [the traditional healer] I can sit and talk and explain things for many hours. Sitting here is important for healing. It is necessary to do this if you want to be cured …. I walk here and then return [over the diagram the healer has drawn]. I turn this way and follow his instructions … when I go to Kilifi [hospital], the doctor does not talk to me. He just asks me some few questions and then gives me a paper [prescription]. That is it!*” (Patient with epilepsy)

#### 5.2.5 Levels of stress

Reduction in stress for the PWE and their families could be reduced by further education about the causes, intermittent nature and treatment of epilepsy, providing a continuous supply of affordable AED that controlled the seizures and, also, psychosocial support. The latter required time and was often better supplied by traditional healers than health care workers. Psychological strategies may also help [24], and promising preliminary results were documented in Nigeria [25].

## 6 Conclusion

Manderson emphasizes that although research into community perceptions of illness, including local taxonomies, is useful in the design of interventions, a more in-depth understanding of the illness and its social and cultural dimensions is more useful in highlighting barriers to behavior change and how interventions can be both effective and sustainable [26]. The process we used here was an attempt to understand these complexities. Panter-Brick and colleagues say that it is now widely accepted that to be effective, interventions should build upon local existing practices, identify and target the most receptive community members, bolster local skills and priorities, recognize constraints (time, financial, cognitive and social) on human agency and feature mobilization of the community [27]. We believe that the process we used helps identify interventions that are more likely to be effective and sustainable.

## Figures and Tables

**Table 1:** Criteria for sample selection for focus group discussions.

Key informant group	Selection factors	Method of identification
Children with epilepsy (14–18 years)	• Have/have never received available treatment	• Identified from KHDSS
• Have received and rejected available treatment
• Attending school/not attending school
Adults with epilepsy	• Have/have never received available treatment	• Identified from KHDSS
• Have received and rejected available treatment
• Less educated^a^/more educated^b^
Parents of children with epilepsy	• Child has/has never received available treatment	• Identified via the child's record on the KHDSS
• Child has received and rejected available treatment
• Less educated^a^/more educated^b^
Other family members of children living with epilepsy	• Grandmothers of children with mild and severe epilepsy	Identified via the child's record on the KHDSS
• Siblings (aged 14–18 years) of children with mild and severe epilepsy

Severe epilepsy was defined as more than one seizure per week; mild epilepsy was defined as less than one per month;KHDSS: Kilifi Health Demographic Surveillance System.

a Less educated residents indicated 8 years or less of schooling.

b More educated residents indicated more than 8 years of schooling.

**Table 2:** Data collection methods and key informant groups.

Key informant group	Data collection method		
Focus group discussions (no. of participants in parentheses)	Individual interviews	Participatory workshops (no. of participants in parentheses)
Adults with epilepsy	1 (3)	1	1 People with epilepsy (11)
Children with epilepsy	1 (6)	1	1 People with epilepsy (1)
Family members of children with epilepsy	1 Mothers (5)	2 Mothers	1 Parents and grandparents (14)
1 Fathers (3)	1 Father	1 People with epilepsy (2 grandparents, 2 parents and 1 sibling^a^)
2 Siblings (4 and 3)	1 Grandmother	
1 Grandmothers (3)		
Biomedical service providers	2 Community health workers (CHWs) (8 and 10)	2 Dispensary nurses	1 Traditional healers and CHWs (7 CHWs)
2 Clinical officers (private clinic)	1 Biomedical service providers and CHWs (6 CHWs and 1 nurse^b^)
1 Psychiatrist (government hospital)	
1 Pediatrician (government hospital)	
Traditional service providers		3 Traditional healers	1 Traditional healers and CHWs (3)
Community intervention organizations		1 Chairlady of ‘Maendeleo ya wanawake’ (women's organization)	
1 Member of staff at APDK (organization for the rehabilitation of people with disabilities)
Units of analysis (number of people)^c^	9 (45)	17 (17)	4 (48)

a Although this participatory workshop was intended for people with epilepsy (PWE), 5 family members also accompanied some of the PWE and participated in the workshop.

b We invited other biomedical service providers to this workshop, but all except one nurse said they were too busy to attend.

c Total units of analysis = 30; total number of people involved = 110.

**Table 3:** Components for the education intervention.

Intervention for CHWs, THs and community members	Intervention for biomedical providers
• What is epilepsy?	• Epidemiology of epilepsy
• Types of seizures	• Definition of epilepsy, seizures and other terminologies
• Causes of epilepsy	• Causes of epilepsy
• Effects of epilepsy on child development	• Common precipitating factors of epilepsy
• Treatment of epilepsy: what drugs can and cannot do?	• International classification of epileptic seizures
• Side effects of drugs	• Diagnosis of epilepsy
• Drug safety	• Differential diagnosis of epilepsy
• What to do and not to do during a seizure	• Conditions co-existing with epilepsy
• When to take PWE to hospital	• Management of epilepsy
• Prevention of epilepsy	
• What PWE can and cannot do	
• Advise to families on how to live with PWE	
